# Balloon angiopLasty for intracranial Atherosclerotic minor Stroke/TIA (BLAST): study protocol for a multicenter prospective cohort study

**DOI:** 10.3389/fneur.2024.1385546

**Published:** 2024-05-24

**Authors:** Shuang Qi, Liang Liu, Fei-Xue Yue, Jing Qiu, Wei Li, Chao Li, Thanh N. Nguyen, Ming Wei, Hui-Sheng Chen, Shou-Chun Wang

**Affiliations:** ^1^Department of Neurology, First Affiliated Hospital of Jilin University, Changchun, China; ^2^Department of Neurology, General Hospital of Northern Theatre Command, Shenyang, China; ^3^Neurology, Radiology, Boston Medical Center, Boston, MA, United States; ^4^Department of Neurosurgery, Tianjin Huanhu Hospital, Tianjin, China

**Keywords:** submaximal balloon angioplasty, intracranial atherosclerotic stenosis, ischemic stroke, transient ischemic attack, medical therapy

## Abstract

**Rationale/Aim:**

Intracranial atherosclerotic stenosis (ICAS) is a common cause of stroke in Asia and is significantly associated with stroke recurrence. The Balloon angiopLasty for intracranial Atherosclerotic minor Stroke/TIA (BLAST) study aims to evaluate the safety and effectiveness of early submaximal balloon angioplasty (SBA) combined with standard medical therapy vs. standard medical therapy alone in patients with minor stroke or transient ischemic attack (TIA) due to ICAS.

**Methods:**

The BLAST study is a multicenter prospective cohort study which will enroll patients with minor stroke or TIA due to symptomatic ICAS within 1 week of symptom onset from 20 centers in China. Eligible patients will receive either SBA with standard medical therapy or standard medical therapy alone based on the decision of the patient or legal representative. Participants will be followed up for 1 year.

**Study outcomes:**

The primary outcome is a composite of stroke or death within 30 days or ischemic stroke in the culprit artery territory from 30 days to 1 year. Secondary outcomes include stroke or death within 30 days, ischemic stroke in the culprit artery territory from 30 days to 1 year, restenosis rate of the culprit artery at 1 year, and neurological improvement at 90 days (assessed by mRS score). Safety outcomes include intracranial hemorrhage within 30 days and endovascular complications.

**Sample size estimate:**

According to previous studies, the incidence of the composite clinical outcomes is 15% in the group receiving medical therapy alone. We assumed the incidence would decrease to 5% in the SBA combined with the medical therapy group. The target sample size is 416 patients (208 per group), with 90% power and 5% type I error, allowing for a 10% loss to follow-up.

**Implications:**

The BLAST study will provide evidence regarding whether early SBA can reduce stroke recurrence and mortality in patients with minor stroke/TIA due to ICAS compared with medical therapy alone.

**Clinical trial registration:**Clinicaltrials.gov, NCT06014723.

## Introduction

In China, stroke is the leading cause of death, and its burden is substantial (1). Intracranial atherosclerotic stenosis (ICAS) is a common cause of stroke and transient ischemic attack (TIA) worldwide, particularly in Asia (2–5). The recurrence rate of stroke caused by ICAS within 1 year was found to be as high as 20% (6–8). Therefore, preventing recurrence after a stroke with ICAS is an important area for study.

Over the past 20 years, several trials have investigated stent placement combined with medical therapy in this population; however, the results have been neutral, and the risk of recurrent stroke remains high (9–11). Compared with stenting, submaximal balloon angioplasty (SBA) could be a safer and simpler intervention that may be an effective strategy for preventing recurrent stroke (12, 13). For example, prior meta-analysis found that the incidence of stroke or death within 30 days after SBA was 3–4.9% (14, 15), and the one-year stroke occurrence rate was 5% (15), which was lower than that of stent placement (9–11).

Minor stroke, defined as a National Institutes of Health Stroke Scale (NIHSS) score ≤ 5, accounted for about half of acute ischemic stroke patients (16, 17). There is high risk of early recurrence of stroke in this population, especially caused by ICAS although enhanced medical treatment was administrated (18, 19). To date, no systematic investigation has compared the effectiveness of SBA combined with standard medical therapy vs. standard medical therapy alone in patients with minor stroke/TIA with ICAS. Therefore, we design a multicenter prospective cohort study to evaluate the safety and effectiveness of early SBA combined with medical therapy vs. medical therapy alone in minor stroke/TIA patients with ICAS.

## Methods

### Study design

The Balloon angiopLasty for intracranial Atherosclerotic minor Stroke/TIA (BLAST) study is a multicenter prospective cohort study designed to investigate the safety and effectiveness of early SBA combined with medical therapy vs. medical therapy alone in patients with minor stroke/TIA with ICAS. This study was approved by the Ethics Committee of the First Hospital of Jilin University (NO.23K081) and other participating hospitals. The study was registered at ClinicalTrials.gov (NCT06014723).

### Target population

**Figure 1:**
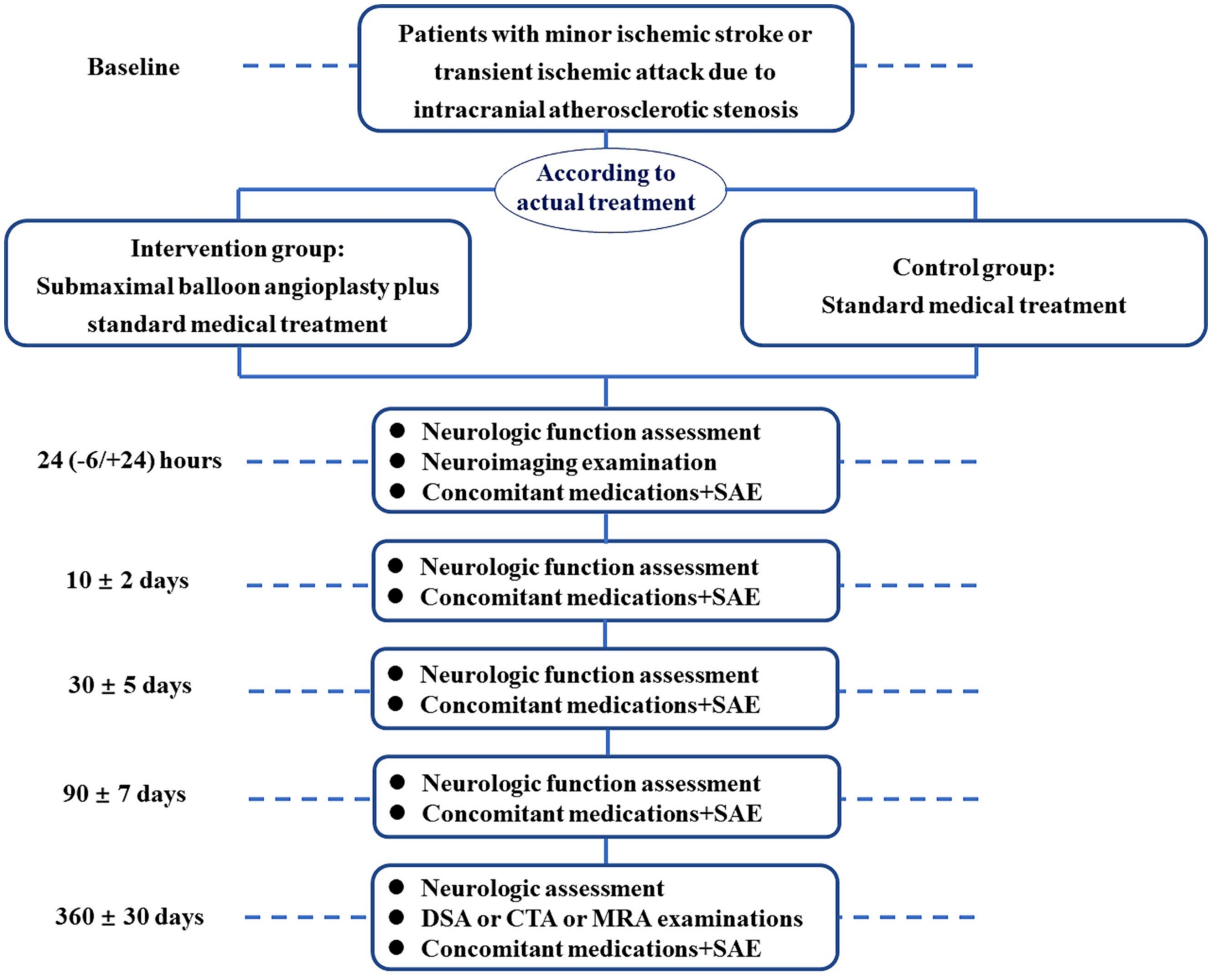
Patient enrollment flowchart. SAE, severe adverse events; DSA, digital subtraction angiography; CTA, computed tomography angiography; MRA, magnetic resonance angiography.

A total of 416 patients from 20 hospitals in China will be enrolled and allocated to either the SBA group (*n* = 208) or the control group (*n* = 208) based on patient treatment preferences. These centers are chosen based on high-capacity centers with neurointervention and good cooperation experience. Eligible patients are adults aged 30–80 years with symptomatic ICAS (70–99% stenosis) resulting in minor stroke (NIHSS score ≤ 5) within 1 week of onset or from symptom exacerbation (NIHSS score increased by ≥2 points) or intermediate- to high-risk TIA, defined as ABCD2 score ≥ 4 within 1 week of the last symptom episode based on radiologic and clinical presentation. Eligible lesions include the intracranial segment of the internal carotid artery (segments C4–C7), the M1 segment of the middle cerebral artery, the V4 segment of the vertebral artery, and the basilar artery. The main exclusion criteria include allergy to contrast media non-atherosclerotic disease-related stenosis; penetrating branch lesion ipsilateral to the target lesion; presence of severe stenosis of the extracranial segment on the side of the target lesion; previous endovascular treatment of the ipsilateral vessel; presence of intracranial aneurysms, tumors, and vascular malformations; any form of ipsilateral intracranial hemorrhage within 3 months and contralateral intracranial hemorrhage within 2 weeks; presence of atrial fibrillation, severe heart dysfunction, liver or kidney dysfunction; patients with tumors and serious diseases whose expected survival time is ≤1 year; and others. The detailed inclusion/exclusion criteria are listed in Table 1. Before enrolling the first patient, all participating centers will receive standard training on the study protocol, inclusion/exclusion criteria, and other relevant details. Patients who meet the inclusion criteria and those who do not meet the exclusion criteria will be eligible to participate (Figure 1). All patients and their legal representatives will be required to sign informed consent.

**Table 1:** Inclusion and exclusion criteria.

**Inclusion criteria**
1.age range of 30–80 years;
2.symptomatic ICAS (C4–C7 segments of ICA, M1 segment of the MCA, V4 segment of the VA, and BA) with 70–99% stenosis confirmed by DSA;
3.minor stroke (NIHSS score ≤ 5) within 1 week of onset or from symptom exacerbation (NIHSS score increased by ≥2 points) or intermediate- to high-risk TIA, defined as ABCD^2^ score ≥ 4 within 1 week of the last symptom episode;
4.diameter of the culprit artery ranging from 2.0 to 4.5 mm, and the length of the stenosis≤14 mm;
5.mRS ≤ 2 before endovascular treatment;
6.no large ischemic region found on CT or MRI (ASPECTS ≥ 6 or pc-ASPECTS ≥ 8);
7.written informed consent obtained from the patient or legally responsible person.
**Exclusion criteria**
1.allergy to contrast media;
2.non-atherosclerotic disease-related stenosis, such as arterial dissection, Moya-Moya disease, and arteritis;
3.penetrating branch lesion ipsilateral to the target lesion (infarction caused by penetrating branch occlusion is defined as stenosis of the MCA or BA, but only with simple basal ganglia or brainstem/thalamic infarction);
4.presence of severe stenosis of the extracranial segment on the side of the target lesion;
5.previous endovascular treatment of the ipsilateral vessel;
6.presence of intracranial aneurysms, tumors, and vascular malformations;
7.any form of ipsilateral intracranial hemorrhage within 3 months and contralateral intracranial hemorrhage within 2 weeks;
8.resence of atrial fibrillation, severe heart dysfunction, liver or kidney dysfunction; patients with tumors and serious diseases whose expected survival time is ≤1 year;
9.hemoglobin ≤100 g/L, platelet count ≤100 × 10^9^/L, INR > 1.5 (irreversible), coagulopathy or irremediable bleeding factors;
10.uncontrollable hypertension: systolic blood pressure > 185 mmHg and/or diastolic blood pressure > 110 mmHg;
11.poor glycemic control (random blood glucose >22.2 mmol/L);
12.history of major surgery within 30 days prior to enrollment or surgery plan within 90 days after enrollment;
13.pregnancy or lactation;
14.other conditions that the researchers think make the patient unsuitable for the study.

ICAS, intracranial atherosclerotic stenosis; ICA, internal carotid artery; MCA, middle cerebral artery; VA, vertebral artery; BA, basilar artery; ASPECTS, Alberta Stroke Program Early CT Score; pc-ASPECTS, posterior circulation ASPECTS; DSA, digital subtraction angiography; NIHSS, National Institutes of Health Stroke Scale; TIA, transient ischemic attack; mRS, modified Rankin Scale; CT, computed tomography; MRI, magnetic resonance imaging; INR, international standardized ratio.

### Treatment strategy

#### The SBA group

In the SBA group, patients will undergo SBA within 1 week of stroke or TIA onset on the basis of standard medical therapy (20). SBA aims to improve stenosis of the culprit artery by more than 20%.

The procedure involves the following steps: (1) Under general or local anesthesia, femoral artery access is obtained, and heparinization is performed (trans-radial access may be used for posterior circulation, aortic arch or abdominal aortic tortuosity); (2) A 6–8F guide catheter is selected per operator preference (an access catheter may be used if the anatomy is tortuous); (3) The diameter of the stenosis, diameter of the artery proximal and distal to the artery is measured; (4) An 0.014 microwire is passed through the lesion into the distal branch of the artery, with microcatheter assistance as needed; (5) The balloon with a diameter of 50–80% of the normal vascular diameter is advanced over the microwire to the lesion, and is slowly inflated (1 atm/min) for 30–120 s, then deflated. If no major dissection or rupture is observed, the balloon is withdrawn while the wire remains in place. Intraprocedural antiplatelet therapy (glycoprotein IIb/IIIa receptor antagonist) may be administered as needed; (6) Angiography is performed after balloon removal to assess the stenosis. If the stenosis is improved by >20% without major dissection, repeat angiography is done within 10–15 min. If the improvement is maintained, the procedure is complete; otherwise, up to 3 repeat dilatations may be performed.

Rescue stenting may be performed if the following occurs: (1) After SBA, residual stenosis remains>80% or forward flow is unstable with a modified Thrombolysis in Cerebral Infarction (mTICI) score < 2b; (2) local dissection causes unstable distal flow and mTICI < 2b.

#### The control group

In the control group, patients will receive standard medical therapy based on current guidelines (20). The antithrombotic regimen will be aspirin 100 mg daily for the duration of follow-up and clopidogrel 75 mg daily for 90 days after enrollment, with continuation determined by the local investigator. For clopidogrel-resistant patients, the investigator may switch to ticagrelor. Lipid-lowering therapy with statins or PCSK9 inhibitors will achieve a low-density lipoprotein target of <1.8 mmol/L (70 mg/dL). Blood pressure will be maintained at <140/90 mmHg.

Timely and positive control of risk factors in both groups will include smoking cessation, a low-salt, low-fat diet, and appropriate exercise.

In the prospective cohort study, eligible patients will receive either SBA with standard medical therapy or standard medical therapy alone based on the decision of the patient or legal representative.

### Outcomes

The primary outcome is a composite of stroke (hemorrhagic and ischemic), or death within 30 days, or ischemic stroke in the culprit artery territory from 30 days to 1 year. Secondary outcomes will include stroke (hemorrhagic and ischemic) or death within 30 days, ischemic stroke in the culprit artery territory from 30 days to 1 year, restenosis of the culprit artery at 1 year defined as exceeding 50% of the stenosis degree or narrowing reaching or surpassing the stenosis degree at the time of the procedure, and neurological functional outcome at 90 days (assessed using the mRS score). Safety outcomes include intracranial hemorrhage within 30 days and endovascular complications. Follow-up will occur at 24 h, discharge, 30 days, 90 days, and 1 year after enrollment. All clinical outcomes including stroke and mRS will be evaluated by certified assessors who are blinded to allocation treatment.

### Data management and quality control

All data will be recorded in the patient’s case report form. The independent Data Safety and Monitoring Committee (iDSMC) will check the data for safety reasons. The iDSMC reserves the right to terminate the study prematurely if unexpected high rates of complication or ICH rates in SBA group during the research. A central core laboratory for imaging is created to judge intracranial hemorrhage events (ECASS definition) and assess neuroimaging data. The protocol is designed by Hui-Sheng Chen and Shou-Chun Wang, and is discussed by the trial steering committee. The trial steering committee consists of external scientific advisors who will regularly monitor the research and data. The trial steering committee will organize teleconferencing or physical meetings to provide recommendations regarding the study.

### Sample size estimates

Based on previous studies, the 1-year incidence of the primary outcome is estimated to be 15% with medical therapy alone (10, 21). We hypothesize this would decrease to 5% with SBA combined with medical therapy (15). With 90% power and an alpha value of 0.05 (two-sided), the sample size required to test superiority is 368. Accounting for a 10% loss to follow-up and alpha spending for the interim analysis (O’Brien-Fleming), the total sample size is 416. Thus, 416 patients will be enrolled (208 per group).

### Data analysis

The baseline patient characteristics will be presented in both groups. Categorical variables will be expressed as frequencies and percentages. Chi-square tests will be used to compare differences between groups. Continuous variables will be presented as mean ± standard deviation or median and quartiles after testing for normality using the Kolmogorov–Smirnov test. The Student’s *t*-test or the Mann–Whitney U test will compare group differences based on normality. Missing data will be handled using multiple imputations.

Primary and secondary outcomes will be analyzed using linear or Cox regression, as appropriate, after adjusting for key prognostic factors (age, sex, baseline NIHSS score, and pre-stroke mRS score, and presention with TIA vs. stroke). Statistical analysis will be performed using SPSS 24.0, with two-sided *p* < 0.05 considered statistically significant.

The primary endpoint will further be stratified by age (≤65 vs. >65 years), gender (male vs. female), hypertension (yes vs. no), diabetes mellitus (yes vs. no), smoking (yes vs. no), baseline SBP (<140 vs. ≥140 mmHg), clinical presentation (TIA vs. stroke), mechanism of stroke (i.e., borderzone vs. perforator vs. embolic), lesion location (anterior circulation vs. posterior circulation), whether the patient is already on pre-stroke aspirin or DAPT.

### Current status

This study was registered online on August 25, 2023. At the time of the first submission of this manuscript, 40 patients had been recruited. Recruitment will continue until the target sample size is reached by December 2026.

## Discussion

The BLAST study aims to evaluate the safety and effectiveness of early SBA combined with medical therapy compared to medical therapy alone in patients with minor stroke or TIA. The study population will comprise of patients with symptomatic ICAS (70–99% stenosis) resulting in minor stroke or TIA within 1 week after symptom onset.

No clinical trial has demonstrated the clear benefit of stenting over medical therapy alone for stroke caused by intracranial atherosclerosis. The SAMMPRIS trial, the first randomized controlled trial of stenting combined with medical therapy versus medical therapy for ICAS, was stopped early because of the higher 30-day stroke and death rate with stenting (14.7% vs. 5.8%, *p* = 0.002) (9). Similarly, the VISSIT study found higher 30-day recurrence after stenting (24.1% vs. 9.4%, *p* = 0.05) (10). More recently, the CASSISS study in China showed no difference in short or long-term recurrence between stenting plus medical therapy and medical therapy (8.0% vs. 7.2%, *p* = 0.82) (11). There are two potential explanations for these disparate results. First, a wide range of patients were enrolled. In the SAMMPRIS trial, patients had non-disabling stroke/TIA within 30 days (median, 7 days from onset to treatment) (9). The VISSIT study enrolled patients with TIA or stroke within 30 days of onset, with a median time of 9–15 days between onset and treatment (10). In the CASSISS study, patients experienced symptoms within 21 days (median, 35 days of treatment) (11). We contend that the benefit of an endovascular intervention decreases with longer onset-to-treatment time since prior studies have shown that carotid revascularization have the best outcomes within 2 weeks of symptoms (22).

Another potential reason to explain the lack of benefit of endovascular therapy may be the complexity of stent implantation and the relatively high procedural complications, leading to bias due to heterogeneity in operator experience and patient selection. Unlike stenting, balloon dilation is a simpler procedure, and a smaller balloon may prevent the disruption of fragile plaques. This could reduce complications, such as dissection, rupture, occlusion, thromboembolism, and perforator occlusion. An RCT comparing balloon dilatation plus aggressive medical management with aggressive medical management for symptomatic ICAS is underway (23). A meta-analysis reported a 4.9% rate of stroke/death within 30 days of SBA for ICAS (14). Another meta-analysis showed stroke rates after SBA for ICAS of 3% at 30 days and 5% at 1 year (15). The incidence rate was lower than that reported in previous studies (SAMMPRIS, 14.7% within 30 days and 19.7% within 1 year; VISSIT, 24.1% within 30 days and 36.2% within 1 year; and CASSIS, 5.1% within 30 days and 8% within 1 year). Previous meta-analyses have also suggested that SBA and medical therapy have similar short-term outcomes; however, SBA may better prevent long-term stroke recurrence (12). Thus, we hypothesized that SBA plus medical therapy may outperform medical therapy alone in patients with minor stroke/TIA with ICAS within 1 week of onset who are at a high risk of recurrence.

The main limitation of this trial is the prospective cohort study, but not randomized control trial, which undoubtedly will weaken the conclusion given the intrinsic flaws of prospective cohort study. Another is that the findings in this trial will be limited to Chinese population, given the differences in etiology and co-morbid factors of ischemic stroke patients between Chinese and other race.

## Summary and conclusion

The BLAST study will provide substantial evidence on whether early SBA reduces 30-day stroke recurrence, mortality, and 1-year ipsilateral ischemic stroke compared with medical therapy alone in patients with minor stroke/TIA caused by intracranial atherosclerosis.

## Ethics statement

The studies involving humans were approved by the Ethics Committee of the First Hospital of Jilin University (NO.23K081). The studies were conducted in accordance with the local legislation and institutional requirements. The participants provided their written informed consent to participate in this study.

## Author contributions

SQ: Writing – original draft. LL: Writing – original draft. F-XY: Writing – original draft. JQ: Writing – original draft. WL: Writing – original draft. CL: Writing – original draft. TN: Writing – review & editing. MW: Writing – review & editing. H-SC: Conceptualization, Supervision, Writing – review & editing. S-CW: Writing – review & editing.
